# Survival Rate in Thalassemia Major Patients: Difference between Date of Diagnosis and Date of Birth as an Index Date for Calculating Follow Up

**Published:** 2018-05

**Authors:** Jafar HASSANZADEH, Alireza MIRAHMADIZADEH, Mehran KARIMI, Shahab REZAEIAN

**Affiliations:** 1. Research Center for Health Sciences, Department of Epidemiology, School of Health, Shiraz University of Medical Sciences, Shiraz, Iran; 2. Dept. of Epidemiology, School of Health, Non-Communicable Diseases Research Center, Shiraz University of Medical Sciences, Shiraz, Iran; 3. Hematology Research Center, Shiraz University of Medical Sciences, Shiraz, Iran; 4. Research Center for Environmental Determinants of Health, Kermanshah University of Medical Sciences, Kermanshah, Iran; 5. Dept. of Epidemiology, School of Health, Shiraz University of Medical Sciences, Shiraz, Iran

## Dear Editor-in-Chief

Thalassemia is one of the genetic diseases detected before birth by prenatal screening tests which the success of them has been reported (1). In other words, the delay in diagnosis should not have been in the thalassemia prevention program (TPP). Despite over several decades of implementation the TPP, not only there are still a number of new cases (2) but also there are cases with delay in diagnosis (3). Recently a paper was published to determine survival and complication rates in patients with thalassemia major (TM) in Taiwan (4). The date of diagnosis was considered as index date for calculating the duration of follow-up for each patient. Delay in diagnosis is associated with more advanced disease stage which leads to decreased survival (5, 6). Hence, this issue should be mentioned as an important limitation of their study.

**Fig. 1:**
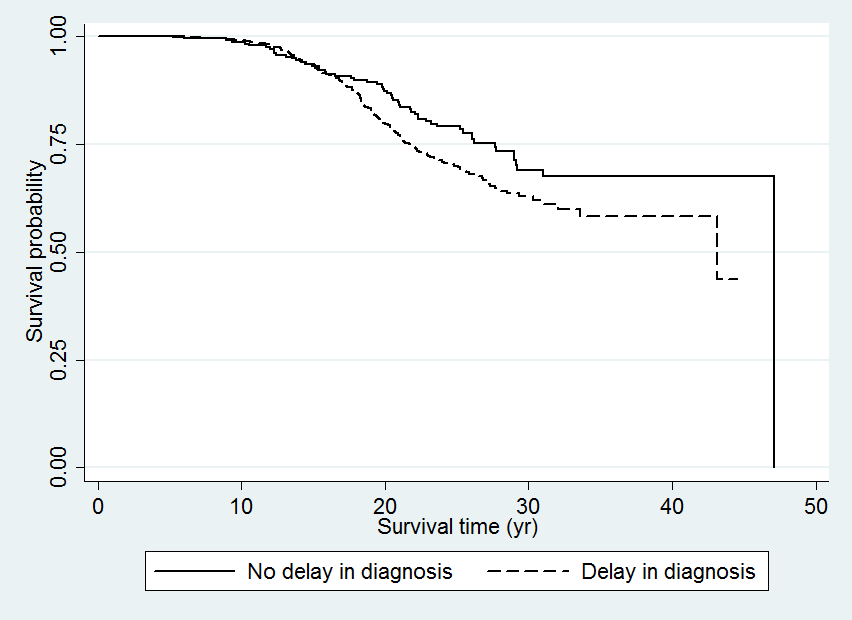
Kaplan-Meier survival curve for the thalassemia major patients by delay in diagnosis date (Log-rank test, *P*=0.027)

In our retrospective cohort study, conducted on 704 Iranian TM patients in 2016, the survival rates were calculated by delay in diagnosis status (no delay or delay in diagnosis). In this TM cohort, 192 patients died. Delayed diagnosis was observed in 460 (65.3%) of the TM patients. Figure 1 showed the Kaplan-Meier survival curves for the patients according to the delay in diagnosis. There was found a significant difference between the survival rates of patients with and without delay in diagnosis (*P*=0.027). We also calculated the attribute fraction among patients with delay in diagnosis and found that about 25% of thalassemia related deaths among the patients with delayed diagnosis can be attributed to delay in diagnosis. In other words, if delay in diagnosis did not occur, about 25% of thalassemia related deaths could be avoided.

We had no information on the causes of delayed diagnosis. However, this issue is related to several factors such as lack of knowledge about being minor, avoiding PND screening tests because of religious beliefs, and screening test errors (7). We supposed if there were not any reason for delay in TM diagnosis, the TPP could have prevented from about 450 new cases.

Delay in TM diagnosis as an independent factor might have impact on the mortality rates of patients.

Improvement of community education and also reduction of the screening tests errors may reduce the incidence of new cases.
